# Temporal trends in Prevalence, Awareness, Treatment, and Control of Hypertension from 2000 to 2010 in Chengdu, China

**DOI:** 10.1038/s41598-017-09579-2

**Published:** 2017-08-21

**Authors:** Gang Huang, Jun-bo Xu, Ya Liu, Zhao-hui Liu, Yun-lan Zhang, Yue-Lei Wu, Rui-lian Wen, Shi Zhu, Ting-jie Zhang

**Affiliations:** 1 Cardiovascular and Metabolic Disease Center, The Second People’s Hospital of Chengdu, Chengdu, China; 2 Department of Cardiology, The Second People’s Hospital of Chengdu, Chengdu, China; 3 Department of Cardiology, The First People’s Hospital of Chengdu, Chengdu, China

## Abstract

This study aimed to describe trends in prevalence, awareness, treatment, and control in hypertension in Chengdu from 2000 to 2010. Two community-based cross sectional surveys were conducted among those aged 40–79 years in 2000 (n = 4850) and 2010 (n = 5456). Demographic characteristics, blood pressure (BP) and associated risk factors were examined. Mean systolic and diastolic BP increased from 117.8 ± 33.9 to 132.1 ± 21.2 mmHg (P < 0.001), and 75.3 ± 19.1 to 79.3 ± 11.2 mmHg (P < 0.001) over past decade, respectively. The prevalence of hypertension increased from 27.7 to 29.4% (P < 0.001). Awareness increased from 37.7 to 42.5% (P < 0.001). The overall treatment rate increased from 20.9 to 28.0% (P < 0.0001), while among subjects aware of hypertension, treatment increased from 46.9 to 65.8% (P < 0.001). In hypertensives, control increased from 6.8 to 6.9% (P = 0.6684). Nevertheless, in hypertensives aware and treated, control decreased from 32.5 to 24.3% (P = 0.020). Hypertension prevalence increased in the last decade, while awareness, treatment and control remained considerably low in Chengdu.

## Introduction

Cardiovascular disease is the leading cause of death in Chinese people aged 40 years and older^1, 2^. In the Chinese population, more than a quarter of adults have two cardiovascular risk factors (i.e., smoking, overweight, hypertension, dyslipidaemia, or hyperglycaemia), and at least 17% adults have three risk factors^3^. As a major risk factor of cardiovascular diseases, hypertension has become a common health challenge with rising prevalence and morbidity in China^4^, of which the overall prevalence is about 30%^5^.

Chengdu is the capital city of Sichuan, which is in southwestern China with a population of 132 million. Accompanied by industrialization and urbanization of Chengdu, unhealthy changes in lifestyle have increased the risk of chronic cardiovascular disease. However, evidence in support of hypertension trend is still not available in Chengdu.

Two epidemiological surveys were conducted from 1998 to 2000 and from 2008 to 2010, separately, in the community population aimed to investigate the impact of lifestyle changes on hypertension and related cardiovascular risk factors. The present study is a retrospective analysis of two surveys on prevalence, awareness, treatment, and control, as well as related risk factors for adults aged 40–79 years in Chengdu over past decade.

## Results

A total of 4850 and 5456 adults were included in two surveys, respectively. Of these included individuals, 243 and 251 (5.0% and 4.6%) refused to accept examination or had missing data. 4607 and 5205 (95.0% and 95.4%) were eligible for analysis, respectively.

### Demographic characteristics and related risk factors

The characteristics and cardiovascular risk factors of all participants are summarized in Tables 1 and 2. More female than male (*P* < 0.001) were included in 2010. Current smokers decreased over the past ten years (*P* < 0.001). And participants with DM increased sharply (*P* < 0.001). Mean BMI increased nearly 4 units in past ten years (*P* < 0.001). And the prevalence of being overweight increased more than 6% (*P* < 0.001). Moreover, the prevalence of being obese doubled over time (*P* < 0.001). Mean levels of total cholesterol(TC), low-density lipoprotein cholesterol(LDL), triglycerides(TG) and fasting plasma glucose (FPG) increased in 2010 compared with those in 2000 (All *P* < 0.001).

**Table 1:** Characteristics of participants in 2000 and 2010.

Characteristics	2000 N = 4607	2010 N = 5205	P Value
Age (m ± s)	55.7 ± 9.1	56.6 ± 10.3	<0.001
40–49 n, (%)	1181 (25.6)	1330 (25.6)	<0.001
50–59 n, (%)	1955 (42.4)	1882 (36.2)	<0.001
60–69 n, (%)	1069 (23.2)	1331 (25.6)	0.007
70–79 n, (%)	402 (8.7)	662 (12.7)	<0.001
**Gender**			
Male n, (%)	3014 (65.4)	2033 (39.1)	<0.001
Female n, (%)	1593 (34.6)	3172 (60.9)	
**Smoking**			
Never n, (%)	2908 (63.1)	3334 (64.1)	<0.001
Previous n, (%)	585 (12.7)	634 (12.2)	<0.001
Current n, (%)	1114 (24.2)	1237 (23.8)	0.029
**Drinking**			
Never n, (%)	3643 (79.1)	4160 (79.9)	<0.001
Previous n, (%)	286 (6.2)	89 (1.7)	<0.001
Current n, (%)	678 (14.7)	956 (18.4)	<0.001
SBP (mmHg)	117.8 ± 33.9	132.1 ± 21.2	<0.001
DBP (mmHg)	75.3 ± 19.1	79.3 ± 11.2	<0.001
PP (mmHg)	43.3 ± 23.1	52.9 ± 15.4	<0.001

SBP, systolic blood pressure; DBP, diastolic blood pressure; PP, Pulse pressure.

Data are presented as mean ± standard deviation (m ± s) for continuous variables and as frequencies (percentages) for categorical variables. Statistical comparisons are from Pearson chi square tests for categorical variables and one way ANOVA for continuous variables.

**Table 2:** Cardiovascular risk factors in 2000 and 2010.

	2000 n = 4607	2010 n = 5205	*P* Value
DM, n(%)	179 (3.3)	634 (11.3)/287 (5.1)^a^	<0.001/<0.001
BMI, kg/m^2^	19.8 ± 9.2	23.8 ± 3.3	<0.001
Overweight, n(%)	1052 (22.8)	1543 (29.6)	<0.001
Obesity, n(%)	87 (1.9)	223 (4.3)	<0.001
WC, (cm)	76.6 ± 12.3	82.9 ± 5.4	<0.001
TC, (mmol/L)	4.2 ± 1.0	4.6 ± 0.9	<0.001
TG, (mmol/L)	1.5 ± 1.3	1.5 ± 1.0	<0.001
LDL-C, (mmol/L)	2.2 ± 0.4	2.6 ± 0.8	<0.001
HDL-C, (mmol/L)	1.4 ± 0.5	1.4 ± 0.3	<0.001
FPG, (mmol/L)	5.1 ± 1.6	5.6 ± 1.8	<0.001
Uric acid, (μmol/L)	332.5 ± 85.4	305.3 ± 73.6	<0.001
Creatinine, (ml/dL)	75.1 ± 36.5	81.1 ± 20.8	<0.001

BMI, body mass index; DM, diabetes mellitus; FPG, fasting plasma glucose; HDL-C, high density lipoprotein cholesterol; LDL-C, low-density lipoprotein cholesterol; TC, total cholesterol; TG, triglycerides; WC, waist circumference.

Data are presented as mean ± standard deviation (m ± s) for continuous variables and as frequencies (percentages) for categorical variables. Statistical comparisons are from Pearson chi square tests for categorical variables and one way ANOVA for continuous variables. Diabetes was diagnosed by fast glucose in 2000 and by the oral glucose tolerance test in 2010.

^a^ The prevalence of diabetes according to the fast glucose in 2010 was 5.1%.

### Trends in BP over ten years

**Figure 1:**
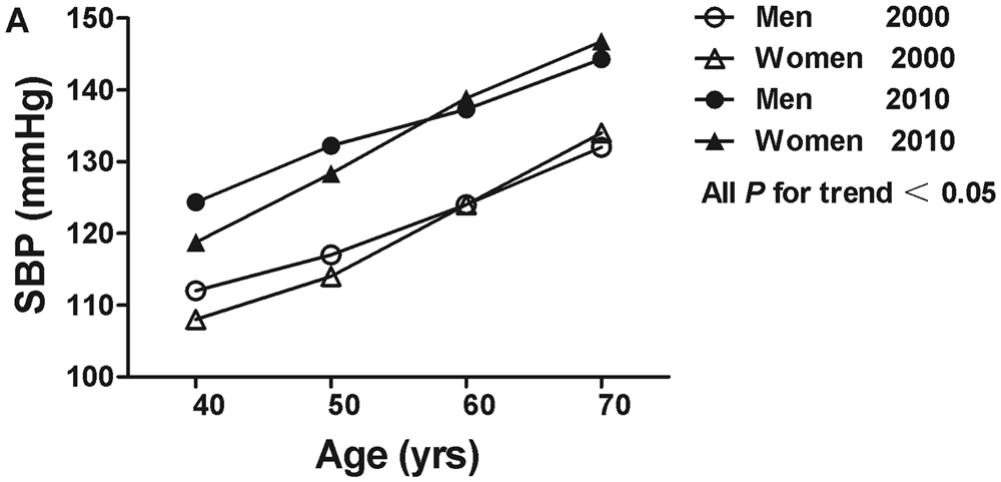
Mean systolic blood pressure (SBP) across age groups by sex in 2000 and 2010.

**Figure 2:**
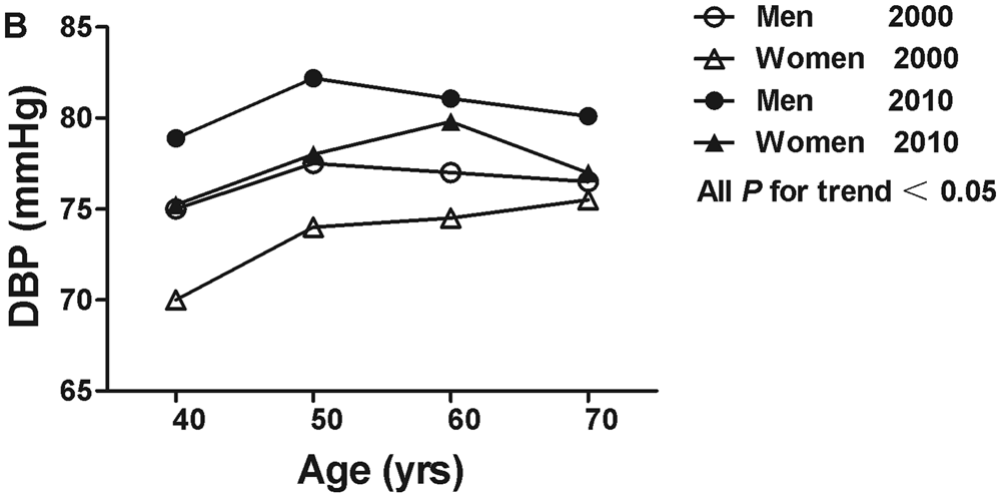
Mean diastolic blood pressure (DBP) across age groups by sex in 2000 and 2010.

Both mean SBP and DBP increased over ten years (All *P* < 0.001). The mean levels of SBP/DBP were 117.8 ± 33.9/75.3 ± 19.1 mmHg (119.6 ± 38.8/76.8 ± 19.3 mmHg for men, 116.9 ± 31.0 mmHg/73.2 ± 18.2 mmHg for women) in 2000, and 132.1 ± 21.2/79.3 ± 11.2 mmHg (133.6 ± 20.4/80.8 ± 11.3 mmHg for men, 130.6 ± 22.0/77.7 ± 11.0 mmHg for women) in 2010, respectively. Mean levels of SBP and DBP were higher in 2010 than it in 2000 by age and sex (All *P* < 0.001). In both years, there was a trend that the levels of SBP and DBP were increasing with age (All *P* for trend < 0.05, Figs 1 and 2). Moreover, mean level of SBP in women aged 60 years and older was higher than that in men in both years. The highest DBP level in men was in the 50–59 year age group in both years, while in women, the highest DBP level was in the 70–79 year age group in 2000 and in the 60–69 year age group in 2010 (Figs 1 and 2).

### Trends in prevalence of hypertension and subtypes

The overall age-standardized prevalence of hypertension has increased nearly 2% in this population over past 10 years (*P* < 0.001, Table 3). The overall prevalence of hypertension was significantly higher in men than that in women (30.2% vs 23.4% in 2000, *P* < 0.001, and 31.0% vs 27.8% in 2010, *P* = 0.022, Table 3). In both surveys, the overall hypertension prevalence increased with age (Both *P* for trend < 0.001). In both surveys, the highest hypertension prevalence was among subjects aged 70 years and older.

**Table 3:** Prevalence of hypertension and subtypes in 2000 and 2010.

	2000 Overall, (men, women)	2010 Overall, (men, women)	*P* value for trend
Pre-hypertension, (%)	35.3 (35.3, 32.7)	37.5 (41.7^a^, 33.0)^b^	
40–49 years	30.3 (37.7, 26.7)	37.6 (44.9, 30.0)	
50–59 years	35.2 (35.6,33.2)	39.8 (41.5, 38.1)	<0.001
60–69 years	39.8 (37.6,42.7)	35.9 (38.6, 32.8)	
70- years	30.2 (32.3,27.1)	28.00 (28.2, 27.7)	
Hypertension, (%)	27.7 (30.2, 23.4)^b^	29.4 (31.0^a^, 27.8^a^)^b^	
40–49 years	16.6 (22.3, 12.6)	15.3 (17.6, 12.9)	
50–59 years	24.6 (28.5, 22.8)	30.9 (34.9, 26.8)	<0.001
60–69 years	41.8 (37.7,42.1)	43.8 (38.6, 46.8)	
70– years	51.5 (50.0, 52.9)	60.4 (60.3, 60.5)	
ISH, (%)	7.5 (6.3, 9.8)^b^	15.1 (13.9^a^, 16.3^a^)	
40–49 years	2.0 (1.6, 2.5)	5.5 (6.1, 4.9)	
50–59 years	3.4 (2.9, 4.6)	12.84 (11.8, 13.9)	<0.001
60–69 years	13.7 (11.7, 17.7)	24.9 (21.3, 29.0)	
70- years	31.8 (27.6, 39.9)	38.4 (34.5, 42.5)	
IDH, (%)	7.8 (9.9, 4.1)	4.8 (5.7^a^, 3.8^a^)^b^	
40–49 years	11.7 (13.6, 3.2)	5.9 (7.2, 4.6)	
50–59 years	10.0 (11.3, 7.4)	5.1 (6.0, 4.1)	<0.001
60–69 years	5.1 (6.2, 2.9)	3.8 (4.2, 3.4)	
70- years	2.7 (3.1, 2.0)	1.9 (2.8, 1.1)	
SDH, (%)	10.8 (12.3, 8.0)^b^	14.9 (16.6^a^, 13.2^a^)^b^	
40–49 years	6.5 (7.6, 4.8)	8.6 (9.5, 7.8)	
50–59 years	10.6 (11.8, 8.1)	17.5 (20.4, 14.5)	<0.001
60–69 years	18.1 (20.0, 14.2)	20.4 (20.5, 20.3)	
70- years	17.7 (20.4, 12.4)	21.2 (25.1, 17.1)	

ISH, isolated systolic hypertension; IDH, isolated diastolic hypertension; SDH, systolic-diastolic hypertension. Overall rates were standardized by age & sex. Statistical comparisons for the trend of prevalence over age groups are from the Cochran-Mantel-Haenszel test. Comparisons between surveys for total prevalence of subtypes are from Pearson chi square tests. P value for trend are for both years 2000 and 2010.

^a^ *P* < 0.05, comparison between 2000 and 2010 by sex.

^b^ *P* < 0.05, comparison between men and women in surveys.

The overall prevalence of pre-hypertension increased about 2% in past 10 years as well (*P* < 0.001, Table 3). It was different from hypertension that the prevalence was decreasing with age in both surveys (Both *P* for trend < 0.001). And the overall pre-hypertension prevalence was higher among men than women (35.3% vs 32.7% in 2000, *P* = 0.059, 41.7% vs 33.0% in 2010, *P* < 0.001, Table 3). In 2010, the highest pre-hypertension prevalence was in subjects aged 50–59 years, compared subjects aged 60-69 years in survey 2000.

The overall prevalence of isolated systolic hypertension (ISH) in 2010 was double that of the 2000 (Table 3). The overall ISH prevalence increased with age in both surveys. Moreover, in subjects aged 60 years and older, the ISH prevalence increased sharply significantly.

The overall prevalence of IDH in 2010 decreased almost 40% compared with that in 2000 (*P* < 0.001). In both surveys, there was a trend that the prevalence decreased with age. Moreover, the prevalence was the highest among subjects aged 40–49 years in both years and the prevalence in each age group in 2000 was higher than that in 2010 (Table 3).

The overall prevalence of SDH increased about 4% in past decade (10.8% in 2000 vs 14.9% in 2010, *P* < 0.001). In both surveys, the SDH prevalence increased with age and the prevalence of SDH was higher for men than women in each age group (Table 3).

### Changes in constituent ratio

**Figure 3:**
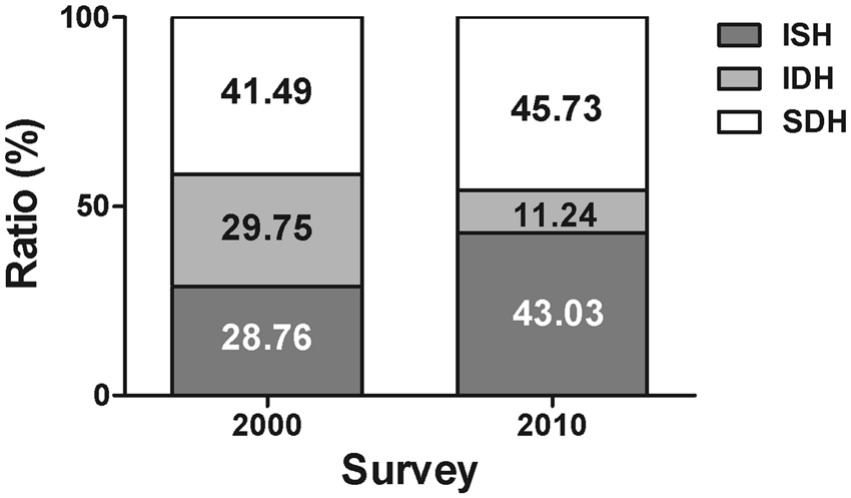
Changes in constituent ratio of hypertension subtypes (ISH = isolated systolic hypertension, IDH = isolated diastolic hypertension, SDH = systolic-diastolic hypertension) over decade.

In 2000, the prevalence of IDH and SDH were higher than that of ISH. However, the prevalence of ISH and SDH were much higher than that of IDH in 2010 (All *P* < 0.001, Fig. 3). Over past 10 years, the prevalence of ISH and SDH increased while IDH prevalence decreased (All *P* < 0.001).

### Trends in awareness, treatment and control of hypertension

The overall awareness slightly increased from 37.7% in 2000 to 42.5% in 2010 (*P* < 0.001). This descent was present in both sexes, and especially noticeable in women (Table 4). While awareness of hypertension was higher among women compared with men. The overall treatment prevalence in overall community population increased nearly 8% over past ten years (*P* < 0.001). The increase was larger in women than in men.

**Table 4:** Prevalence, awareness, treatment, control of hypertension in 2000 and 2010.

	2000 Overall, (men, women)	2010 Overall, (men, women)	*P* Value
Prevalence, (%)	27.7 (30.2, 23.4)	29.4 (31.0^a^, 27.8^a^)	<0.001
Awareness, (%)	37.7 (32.8, 42.2)^b^	42.5 (39.9, 45.3) ^b^	<0.001
Treatment, (%)	20.9 (20.2, 21.7)	28.0 (25.8^a^, 30.4^a^) ^b^	<0.001
Treatment, (%) among awared hypertensives	46.9 (48.5, 45.2)	65.8 (64.7^a^, 67.0^a^)	<0.001
Control, (%)	6.8 (6.4, 7.2)	6.9 (6.0, 7.8)	0.668
Control, (%) among treated hypertensives	32.5 (31.8, 33.3)	24.3 (23.2, 25.5)	0.020

Statistical comparisons between 2000 and 2010 are from Pearson chi square tests.

^a^ *P* < 0.05, comparisons between 2000 and 2010 by sex.

^b^ *P* < 0.05, comparisons between men and women.

Among those who were aware their hypertension, the treatment rose from 46.9% (in 2000) to 65.8% (in 2010) significantly (*P* < 0.001, Table 4). Among overall population who were aware of hypertension, treatment was higher in women than that in men in 2010, while it was lower in 2000. However, the increases of treatment rate among those aware was higher in women than it in men (men: 16.3%, women: 21.9%). Overall control increased only 0.1% with no significance (6.8% to 6.9%, *P* = 0.668) over the past ten years. Among treated hypertensives, control (BP < 140/90 mmHg) was 32.5% in 2000, compare to 24.3% in 2010 (*P* = 0.020, Table 2). Control was also higher in women than in men in both years. Moreover, there was more decreasing of control in men than in women (decreases for men vs women = 8.5% vs 7.8%).

## Discussion

The main findings of this study are as follows: among Chengdu residents aged 40 to 79 years, 1). SBP and DBP levels increase over past decade, 2) prevalence of hypertension and pre hypertension increase steadily with slowly increasing awareness, while treatment and control remain considerably low.

There were more women in survey 2010, mainly because many younger and middle aged men moved to work in more developed east coastal areas (i.e. Guangdong, Fujian, Zhejiang provinces) over past decade. The steadily increased BP level and prevalence may be related to lifestyle changes accompanying with industrialization and urbanization, such as high salt food^6^, rich fat food intake (Sichuan cuisine is characterized by pungent, hot, and salty flavors), and higher BMI^7^. Smoking^8, 9^ and work related stress are associated with hypertension development^10^. Accompanying with the industrialization and urbanization, increased stressful workload may involve in the hypertension prevalence increasing. Limited knowledge of hypertension prevention, diagnosis treatment and its relevant risk factors among community physicians and general population may contribute to the low awareness. Treatment of hypertension was improved with economic development in past decade, especially apparently among those were aware of their hypertension. Higher socioeconomic status could positively improve the awareness and control of hypertension, and people would also have a better compliance of medicine treatment. Accompanying with the rapid economic development of Sichuan, people changed their dietary habits with more unhealthy fatty and salty food, while tended to be more dependent on morden transportation daily. Meanwhile, more people gradually became to care more about their health and generally could relatively afford the disease cost during past decade. It could be one reason for the increasing hypertension prevalence accompanying by increasing awareness and treatment. While during past decade, no apparent control improvement in smoking and drinking, increasing overweight and obesity, and increasing lipid and glucose levels could interact and potentially contribute to the poor hypertension control. Besides, poor compliance with medication and lack of health eduction could also be important reasons for poor BP control.

During past decade, there was no increasing trend of SBP and DBP levels in America^11, 12^ during the past two decades and in Japan^13^, while there was a consistent decreasing of BP in Germany^14^, Spain^15^, Italy^16^ and Korean^17^. From 1988 to 2008, there was an increasing trend of hypertension prevalence in Americans aged 40 years and older^11^, however, the prevalence of hypertension in Japan^13^, Germany^14^, Italy^16^ and Korea^17^ decreased. Other Chinese studies in other areas^18–20^ also have found that there is an increasing trend of prehypertension and hypertension prevalence. A recent study in Chongqing^20^, which is a neighbor city of Sichuan with similar geographic, culture, and dietary background, reported that hypertension prevalence in local adults (23.4% in 2012 and 12.0% in 2002) doubled during past decade. The increasing trend of hypertension prevalence in our study is in accordance with studies in other Chinese cities, especially cities with similar background, and other developing countries, such as India^21^ and Thailand^22^. During the same period, the hypertension awareness and treatment in America^11, 12^, Germany^14^ and Korean^17^ increased substantially, which contributed to a better BP control. And awareness of hypertension in other areas in China tended to increase consistently^18–20^. Nevertheless the treatment in Chengdu is unluckily lower than in Shandong^18^, Beijing^19^, while higher than in Chongqing^20^, India^21^ and Thailand^22^. In past decade, the hypertension control doubled in Germany^14^, Spain^15^ and nearly doubled in America^11^ and it increased steadily in developed couriers, such as Italy^16^ and Korean^17^. Unfortunately, the hypertension control in Chengdu was low, which was in accordance with studies in other Chinese cities^5, 18–20^. It is apparently that improvements in hypertension awareness, treatment contributed to better control in european countries^11, 12, 14–16^.

Lifestyle modification contributing to BP decrease is recommend to general population in Chengdu. People should increase their physical activity, improve levels of fruit and vegetable intake, consume less fatty diet and quit smoking. A better community based primary health care and primary prevention system focused on early diagnosis and treatment, patients management, hypertension knowledge education and lifestyle risk factors modification in general population is needed for better BP control.

This study has following limitations. First, results of this study should be interpreted with caution, since both surveys only focused on population in Chengdu, although main results are comparable to studies in other Chinese cities. Second, the gender constituents in two separate surveys were different and quantity of physical activity was not measured in 2000. Third, due to the different economical support by the government, the methods for diabetes mellitus diagnoses were different in both surveys. Fourth, smoking and alcohol consumption data are based on self-report with the possibility of misclassification of exposure.

In conclusion, hypertension prevalence increased steadily among residents aged 40 to 79 years in Chengdu from 2000 to 2010, while awareness, treatment and control remained considerably low. More efforts should be taken to improve hypertension awareness, treatment and control among middle aged and older residents. Future enhanced public health care program focused on these problems is needed.

## Materials and Methods

### Population

Two cross-sectional surveys were conducted from 1998 to 2000 and from 2008 to 2010 separately in communities of Chengdu, using a multistage cluster sampling. The study population included 4850 and 5456 subjects aged 40–79 years (All subjects were Han ethnicity) living in Chengdu more than 3 years in survey 2000 and survey 2010, respectively. The response rates for the subjects participating in the surveys of 1999 and 2009 were 95.1% and 95.4%, respectively. Ethics approval was obtained from the Ethics Committee of the Second People’s Hospital of Chengdu, China. The methods in the study were in accordance with relevant guidelines and the Declaration of Helsinki. All participants gave informed consent.

### Data collection

Data were collected through a standard household questionnaire interview with additional interviewing and a physical examination in community hospitals in both 2000 and 2010, separately. In both years, all researchers were trained for questionnaire administration, correct cuff size selecting, BP measurement and other techniques needed in surveys with standard techniques. The information collection included demographic characteristics, smoking, alcohol consumption, history of hypertension, cardiovascular disease, diabetes, and administration of antihypertensive medication, physical examination, waist circumference, body weight, and height. In both years, participants had their blood pressure (BP) measurements with a validated mercury sphygmomanometer by trained researchers. Three consecutive BP readings were taken on the right arm with an appropriate cuff size (22 to 26 cm long and 12 to 14 cm wide), in a sitting position after a 10 minutes rest. Participants were not allowed to smoke cigarettes, drink tea/coffee, or do physical exercise half an hour before BP measurements. The room temperature was required to be between 18 °C and 25 °C for measurements. Overnight fasting blood specimens of participants were collected and tested in the central laboratory of our hospital in both years. The oral glucose tolerance test (OGTT) was tested in all subjects in 2010, while not in 2000.

### Definitions

In both years, BP level was defined as the mean value of three measurements and hypertension was defined as systolic blood pressure (SBP) ≥ 140 mmHg, or/and diastolic blood pressure (DBP) ≥ 90 mmHg, and/or normotensives treated with antihypertensive medications. Prehypertension was defined as 120 ≤ SBP < 140 mmHg or/and 80 ≤ DBP < 90 mmHg^23, 24^. Hypertension subtypes in surveys include: isolated systolic hypertension(ISH), defined as 140 mmHg ≤ SBP and DBP < 90 mmHg; isolated diastolic hypertension(IDH), defined as SBP ≤ 140 mmHg and 90 mmHg ≤ DBP; systolic - diastolic hypertension(SDH), defined as 140 mmHg ≤ SBP and 90 mmHg ≤ DBP^23, 24^.

Based on the 1999 World Health Organization standards, diabetes mellitus (DM) was defined as fasting plasma glucose > 7.0 mmol/l (in 2000), and by OGTT (2-h plasma glucose ≥ 11.1 mmol/l, in 2010).

Awareness of hypertension was determined by definite response to question “Have you ever been told that your blood pressure was high or you had a hypertension” by a physician/heath care. Treatment of hypertension was confirmed by saying “yes” to this question, “Are you taking antihypertension medication during last three months, because of your high BP/hypertension?”, Control of hypertension was defined as the measured SBP < 140 mmHg and DBP < 90 mmHg in patients with hypertension under antihypertension treatment^23, 24^.

Body mass index (BMI) was defined as weight (kg)/height (m^2^). Overweight was define as 25 kg/m^2^ ≤ BMI < 28 kg/m^2^. Individuals with a BMI of 30 kg/m^2^ or greater are defined as obese.

### Statistical analysis

Participants were subdivided into 4 age groups: 40 to 49, 50 to 59, 60 to 69, and 70 to 79 years old. Statistical analysis was performed by using of SPSS17.0 for Windows (Chicago, IL, USA). Continuous variables were expressed by mean ± standard deviation. Categorical variables were expressed by percentage (%). Rate of awareness, treatment and control of hypertension were standardized by the China national census. Comparisons between groups used the Chi - Square Test for categorical variables and one way ANOVA for continuous variables. The Cochran - Mantel - Haenszel test was used to analyze for the trend of prevalence over various age groups. A two-sided *P* value of < 0.05 was considered to be statistically significant.
